# Reweighting and validation of the hospital frailty risk score using electronic health records in Germany: a retrospective observational study

**DOI:** 10.1186/s12877-024-05107-w

**Published:** 2024-06-13

**Authors:** Klaus Kaier, Adrian Heidenreich, Markus Jäckel, Vera Oettinger, Alexander Maier, Ingo Hilgendorf, Philipp Breitbart, Tau Hartikainen, Till Keller, Dirk Westermann, Constantin von zur Mühlen

**Affiliations:** 1 Institute of Medical Biometry and Statistics, Faculty of Medicine and Medical Center, University of Freiburg, Hugstetter Str. 49, Freiburg, 79106 Germany; 2 Centre for Big Data Analysis in Cardiology (CeBAC), Department of Cardiology and Angiology, Faculty of Medicine, University of Freiburg, Freiburg, Germany; 3 Medical Centre, Department of Cardiology and Angiology, Faculty of Medicine, University of Freiburg, University Heart Centre Freiburg – Bad Krozingen, University of Freiburg, Freiburg, Germany

**Keywords:** Aged, Machine learning, Supervised learning, Clinical frailty scale, Risk adjustment, Clinical decision making

## Abstract

**Background:**

In the hospital setting, frailty is a significant risk factor, but difficult to measure in clinical practice. We propose a reweighting of an existing diagnoses-based frailty score using routine data from a tertiary care teaching hospital in southern Germany.

**Methods:**

The dataset includes patient characteristics such as sex, age, primary and secondary diagnoses and in-hospital mortality. Based on this information, we recalculate the existing Hospital Frailty Risk Score. The cohort includes patients aged ≥ 75 and was divided into a development cohort (admission year 2011 to 2013, *N* = 30,525) and a validation cohort (2014, *N* = 11,202). A limited external validation is also conducted in a second validation cohort containing inpatient cases aged ≥ 75 in 2022 throughout Germany (*N* = 491,251). In the development cohort, LASSO regression analysis was used to select the most relevant variables and to generate a reweighted Frailty Score for the German setting. Discrimination is assessed using the area under the receiver operating characteristic curve (AUC). Visualization of calibration curves and decision curve analysis were carried out. Applicability of the reweighted Frailty Score in a non-elderly population was assessed using logistic regression models.

**Results:**

Reweighting of the Frailty Score included only 53 out of the 109 frailty-related diagnoses and resulted in substantially better discrimination than the initial weighting of the score (AUC = 0.89 vs. AUC = 0.80, *p* < 0.001 in the validation cohort). Calibration curves show a good agreement between score-based predictions and actual observed mortality. Additional external validation using inpatient cases aged ≥ 75 in 2022 throughout Germany (*N* = 491,251) confirms the results regarding discrimination and calibration and underlines the geographic and temporal validity of the reweighted Frailty Score. Decision curve analysis indicates that the clinical usefulness of the reweighted score as a general decision support tool is superior to the initial version of the score. Assessment of the applicability of the reweighted Frailty Score in a non-elderly population (*N* = 198,819) shows that discrimination is superior to the initial version of the score (AUC = 0.92 vs. AUC = 0.87, *p* < 0.001). In addition, we observe a fairly age-stable influence of the reweighted Frailty Score on in-hospital mortality, which does not differ substantially for women and men.

**Conclusions:**

Our data indicate that the reweighted Frailty Score is superior to the original Frailty Score for identification of older, frail patients at risk for in-hospital mortality. Hence, we recommend using the reweighted Frailty Score in the German in-hospital setting.

**Supplementary Information:**

The online version contains supplementary material available at 10.1186/s12877-024-05107-w.

## Introduction

With the global rise in the elderly population, the fragility of older adults is a significant concern for healthcare systems. Older, frail individuals, in particular, often necessitate additional care and services, leading to a higher likelihood of hospitalization [1]. Frailty typically manifests as a reduced physiological capacity and heightened susceptibility to stressors [2, 3]. Previous studies have shown that frailty correlates with increased mortality rates [4, 5], and considerable economic burdens [6]. As the world ages demographically, it is anticipated that the number of frail individuals will substantially increase [7], emphasizing the need to understand its prevalence. However, there is no consensus on how to assess frailty in clinical settings [8].

Given that frailty significantly influences resource allocation and care planning, its assessment should guide these processes. Yet, identifying frail older individuals faces significant obstacles. Existing tools for measuring frailty exhibit only moderate agreement [9], causing variability in their selection and usage. Moreover, most tools are too complex for acute care settings. Even simpler tools like the Clinical Frailty Scale [10] and Identification of Seniors at Risk [11] require manual assessment, leading to time consumption and potential errors.

Recently, Gilbert et al. introduced the Hospital Frailty Risk Score utilizing International Statistical Classification of Diseases and Related Health Problems, Tenth Revision (ICD-10) codes [12]. This score was developed in a cohort of older patients (aged 75 and older, *n* = 22 139) that were hospitalized between 2013 and 2015 in England. Following the publication of the frailty score in 2018, it attracted considerable attention in the scientific community. This was followed by several external validations of the score in general patient populations [13–17], disease-specific populations [18–21], and even within patients admitted to the intensive care unit [22]. With the exception of intensive care admissions, all of these validations were positive. However, the individual factors contained in the score were not adjusted or reweighted.

In this paper, we propose a reweighting of Gilbert’s score (subsequently referred to as the *original Frailty Score*) using routine data from a single tertiary care teaching hospital in southern Germany. We hypothesize that this reweighted score (subsequently referred to as the *reweighted Frailty Score*) will improve the predictive performance with regard to in-hospital mortality in the German setting. Development of the reweighted Frailty Score takes place using all older patients (aged 75 and older) of the years 2011, 2012 and 2013 (*N* = 30,525) in a German tertiary care teaching hospital. Validation of the reweighted Frailty Score takes place in the same hospital using all older patients of the year 2014 (*N* = 11,202). Furthermore, additional external validation is conducted in a second validation cohort containing inpatient cases aged ≥ 75 in 2022 throughout Germany (*N* = 491,251).

## Methods

The study cohort includes information on all patients hospitalized between 2011 and 2014 at the University Medical Centre Freiburg, a tertiary care teaching hospital in southern Germany [23]. By including all patients of a maximum care provider over several years, it can be assumed that the full range of hospitalized patients in Germany is included. The dataset includes patient characteristics such as sex, age, primary and secondary diagnoses and in-hospital mortality. Diagnoses were coded according to the ICD-10 German modification. Gilbert et al. used a multistep process to identify a total of 109 frailty-related diagnoses on which their Hospital Frailty Risk Score was based. We used the same 109 frailty-related diagnoses to recalculate the Score from Gilbert et al. [12], and to generate a reweighted Frailty Score for the German setting.

The patient cohort was divided into a development cohort (all patients aged 75 years and older hospitalized between 2011 and 2013, *N* = 30,525) and a validation cohort (all patients aged 75 years and older hospitalized in 2014, *N* = 11,202). Furthermore, a second validation cohort was collected using Germany’s Federal Bureau of Statistics (Destatis). The Destatis cohort includes all cases that were hospitalised in Germany in 2022. For reasons of data economy, a random sample of 10% was initially taken and further limited to the inclusion of all patients aged 75 and over (N = 491,251). We were able to request analyses of this cohort, but received only summary results without direct access to individual records. This approach, in line with German law, negates the need for ethics committee approval or informed consent for studies as Destatis ensures data protection by censoring any details that could identify patients or hospitals.

In the development cohort in Freiburg (admission year 2011 to 2013, N = 30,525), variable selection was used to identify the variables relevant for the association with in-hospital mortality. In contrast to previous studies on this topic, variable selection was not based on p-values but on the adaptive lasso [24]. The adaptive lasso is a modification of the standard lasso (Least Absolute Shrinkage and Selection Operator) [25] and is applied using the ‘lasso logit’ command in Stata, a binary logistic regression lasso model. A major advantage of the adaptive lasso is its oracle property, which improves the selection of relevant variables. Unlike other machine learning appraches, the adaptive lasso estimates coefficients that can be directly applied to other settings, enhancing the model’s utility and adaptability. This transferability is crucial for ensuring that the findings are applicable across different settings. The oracle property ensures that as the sample size increases, the adaptive lasso consistently selects the true relevant variables with high probability [24]. In a second step, the penalized coefficients of the model are used to obtain weights for each dichotomous condition. As in Gilbert et al. [12], the regression coefficients were rounded to one decimal point and simply summarized.

Model performance is assessed using the area under the receiver operating characteristic curve (AUC). The AUC is assessed in the development cohort and in the validation cohort. 95% confidence intervals and tests between two AUC ranges are performed using the non-parametric DeLong test [26]. In the development cohort, internal validation was conducted using bootstrap resampling with 1000 samples. We compared bootstrap model discrimination with apparent model discrimination.

In the validation cohort in Freiburg (admission year 2014, *N* = 11,202), calibration plots are constructed as recommended [27]. In Calibration plots, observed mortality was plotted against model predicted mortality with a local regression (loess) smoother fitted across all individuals in the validation cohort to produce a flexible calibration curve [28]. Furthermore, the validation cohort was used to carry out decision curve analysis with the Stata package DCA to assess the net benefit of using the reweighted frailty score, the original frailty score, and the CCI. For prediction models, compared with universal intervention for all or intervention for none, decision curve analysis allows calculation of a ‘net benefit’. The net benefit is equal to the true positives minus the false negatives, weighted by the threshold at which an intervention would be warranted. In our study, the risk thresholds under consideration are equivalent to the mortality risk at which a clinician would recommend that a patient not receive the respective treatment, in accordance with the respective score assessment. Net benefit curves are smoothed using a robust nonlinear smoother.

In the validation cohort in Germany (admission year 2022, *N* = 491,251), we were able to prespecify analyses for the calculating the AUCs, ROC-Curves and calibration plots in accordance to the descriptions above. The application of decision curve analysis, however, was not possible due to technical constraints.

Last but not least, the applicability of the reweighted Frailty Score in a non-elderly population was assessed. Therefore, we used all patients aged 18 years and older hospitalized between 2011 and 2014 in Freiburg, *N* = 198,819 and used logistic regression models to observe the impact of the reweighted Frailty Score on in-hospital mortality across different age groups and the patients sex.

No imputation for missing values could be conducted due to the absence of codes indicating that data were missing. If the patient’s electronic health record did not include information on a clinical characteristic, it was assumed that that characteristic was not present. All analyses were performed using Stata 18 (StataCorp, College Station, Texas, USA).

## Results

In total, *N* = 30,525 and *N* = 11,202 hospitalizations were recorded in the development and validation cohort in Freiburg. In-hospital mortality was 4.12% and 3.85%, respectively, the mean patient’s age was ~ 81 years and ~ 50% of patients were female in both cohorts (see Table 1). According to the weighting proposed by Gilbert et al., the mean original Frailty Score was 4.22 and 4.13 in the development and validation cohort, respectively.

**Table 1:** Patient characteristics in the development cohort in Freiburg (admission year 2011 to 2013, *N* = 30,525) and the validation cohorts in Freiburg (admission year 2014, *N* = 11,202) and Germany (admission year 2022, *N* = 491,251)

*N*	30,525		11,202		491,251	
Age at admission (in years), mean SD	81.01	4.84	80.87	4.95	82.90	5.12
Female sex, %	50.47%		50.56%		54.67%	
Charlson Index, mean SD	2.31	2.75	2.26	2.76	2.12	2.20
Original Frailty Score, mean SD	4.22	5.01	4.13	4.86	6.17	6.25
Reweighted Frailty Score, mean SD	0.21	1.19	0.25	1.23	0.18	1.15
In-hospital mortality, %	4.12%		3.85%		6.08%	

For the reweighting of the Frailty Score, 56 out of 109 comorbidities were removed within the score development process. In the end, only 53 comorbidities were associated with in-hospital mortality risk. Supplemental Table S1 provides an overview over the prevalence of the selected comorbidities in the development and validation dataset. The mean reweighted Frailty Score was 0.21 and 0.25 in the development and validation cohort, respectively (see Table 1).

**Fig. 1:**
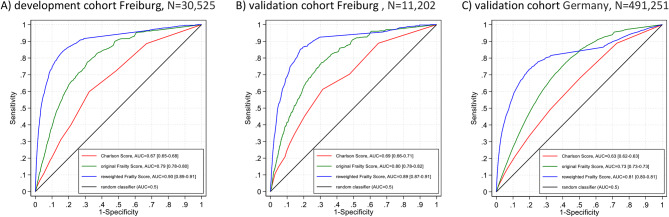
ROC-curves in the development cohort in Freiburg (admission year 2011 to 2013, *N* = 30,525) and the validation cohorts in Freiburg (admission year 2014, *N* = 11,202) and Germany (admission year 2022, *N* = 491,251)

As shown in Fig. 1, reweighting of the Hospital Frailty Risk Score resulted in substantially higher AUCs than the initial weighting of the score. This result is nearly identical in the development (Fig. 1A) and validation cohort (Fig. 1B). In addition, internal validation using bootstrapping shows that overfitting is low, as there is very little difference between the apparent (AUC = 0.90 [0.89–0.91]) and bootstrapped results (AUC 0.89 [0.88–0.90]) in the development cohort (*N* = 30,525).

In the validation cohort in Germany (admission year 2022, *N* = 491,251), patients were slightly older (~ 83 years) and more often female (~ 55%) than in Freiburg. Interestingly, the mean original Frailty Score was higher than in Freiburg, although the Charlson Score and the mean reweighted Frailty Score were comparable (Table 1). In addition, in-hospital mortality was higher across Germany (6.08%) than in the Freiburg cohorts (4.12% and 3.85%). As shown in Fig. 1C, reweighting of the Hospital Frailty Risk Score resulted in substantially better discrimination (AUC = 0.81 [0.80–0.81]) than the initial weighting of the score (AUC = 0.73 [0.73–0.73]) and the Charlson Score (AUC = 0.63 [0.62–0.63]).

**Fig. 2:**
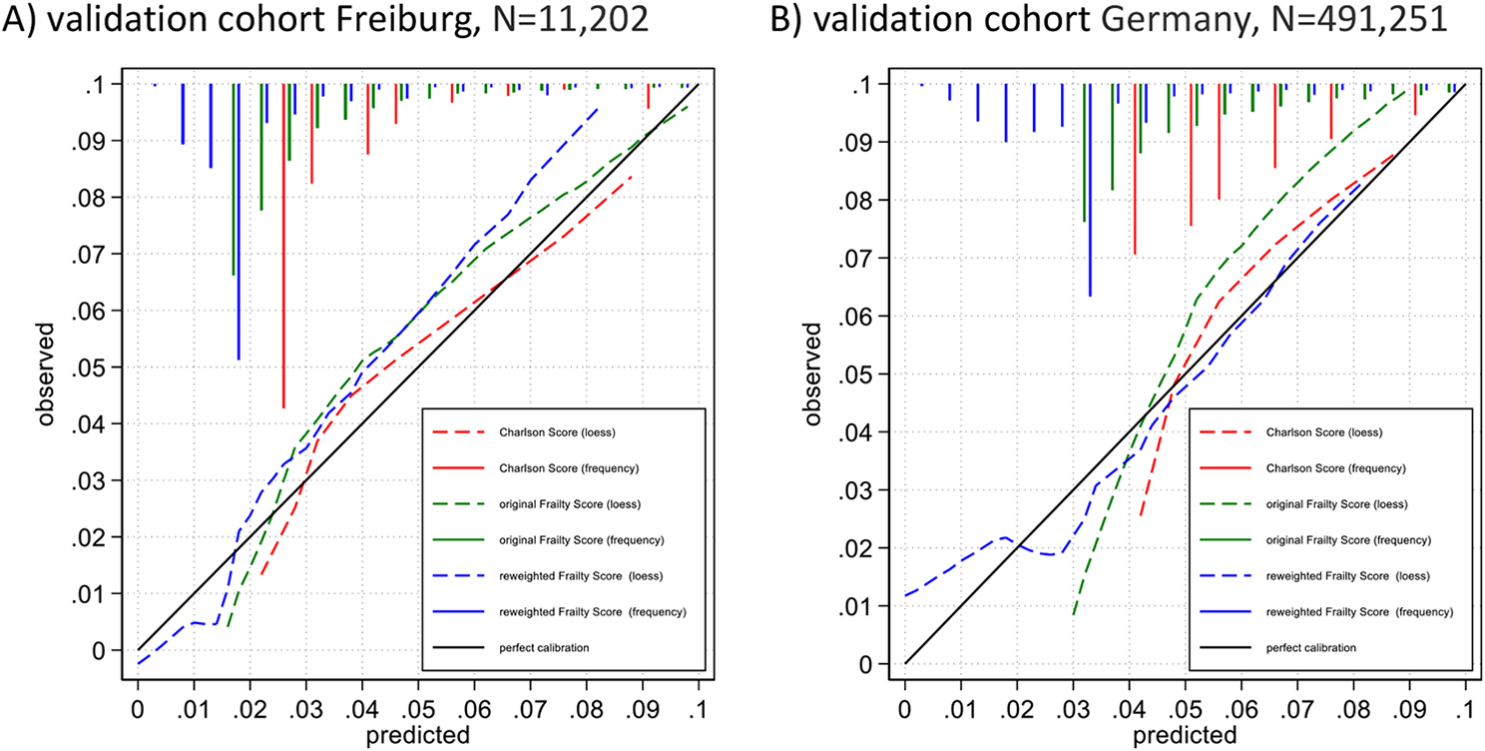
Calibration plots in the validation cohorts in Freiburg (admission year 2014, *N* = 11,202) and Germany (admission year 2022, *N* = 491,251). In the validation cohorts, the observed risk of in-hospital mortality is plotted against the predicted risk from the Charlson Score (red), the original Frailty Score (green) and the reweighted Frailty Score (blue). The solid line represents perfect calibration (with a slope of 1), and the dashed line represents the respective loess smoothed calibration curves. Relative frequencies of the predicted values of the three scores are shown at the top

Calibration of the three scores was compared in the cohorts in Freiburg (admission year 2014, *N* = 11,202) and Germany (admission year 2022, *N* = 491,251) and shown in Fig. 2. Calibration plots show the extent to which the respective scores predict death in patients at low, medium and high risk for in-hospital mortality. Calibration of the Charlson Score and the original Frailty Score lack granularity among the patients with the lowest risk. In the validation cohort in Germany (admission year 2022, *N* = 491,251), the Charlson Score is 0 for a total of 26% and the original Frailty Score is 0 for 17% of the patients. The reweighted Frailty Score, in contrast, contains negative coefficient, and is thus more granular among patients in the lowest risk groups. In Fig. 2, this is particularly shown for patients with a predicted in-hospital mortality risk of < 4%. More detailed calibration plots are presented in the supplemental appendix (Figure S1).

**Fig. 3:**
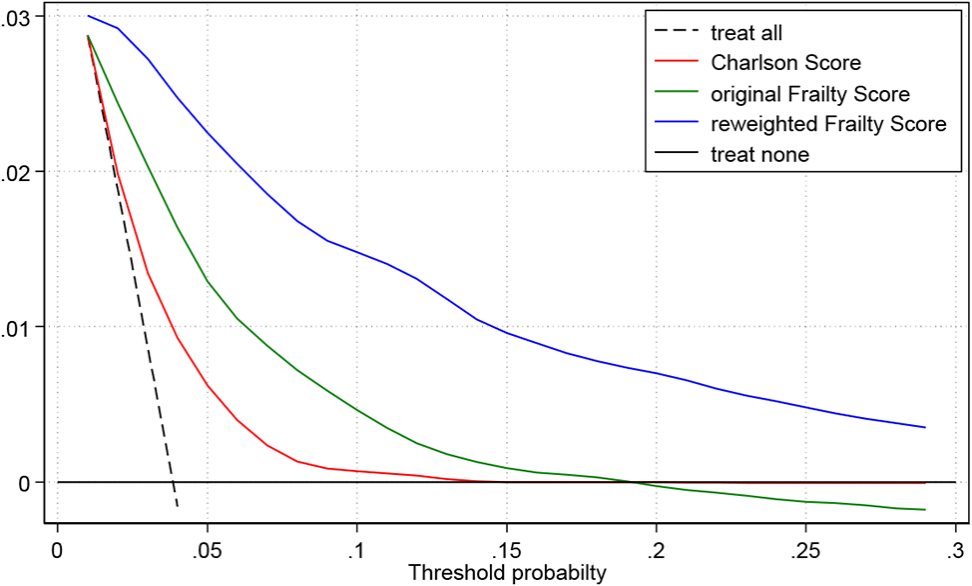
Decision curves in the validation cohort in Freiburg (admission year 2014, *N* = 11,202). Decision curve analysis showing the clinical utility Charlson Score (red), the original Frailty Score (green) and the reweighted Frailty Score (blue) in predicting in-hospital mortality in the validation cohort in Freiburg (*N* = 11,202). The black dashed line represents the net benefit of treating all patients without recognition of any of the three risk scores, assuming that all patients would survive. The black solid line represents the net benefit of refusing treatment for all patients similarly, assuming that all would die after treatment

Figure 3 shows the decision curves demonstrating the clinical usefulness of the three scores as a general decision support tool. Decision curves were constructed using information from the cohort in Freiburg (admission year 2014, *N* = 11,202) to avoid overfitting. It should be noted that there is no single, specific treatment decision in the patient population observed. Instead, there are a multitude of heterogeneous decisions to be made regarding the further clinical course. Often several per hospitalization. The net benefit shown in Fig. 3 describes the clinical benefit resulting from the additional information contained in the risk scores. This can be either the continuation of an existing treatment or the initiation of a new treatment. Overall, the net benefit of the reweighted Frailty Score is superior to that of the original Frailty Score and the Charlson score and the results are consistent across the threshold values observed. This means that the reweighted frailty score can be of significantly greater benefit than the other two scores in any treatment decision.

**Fig. 4:**
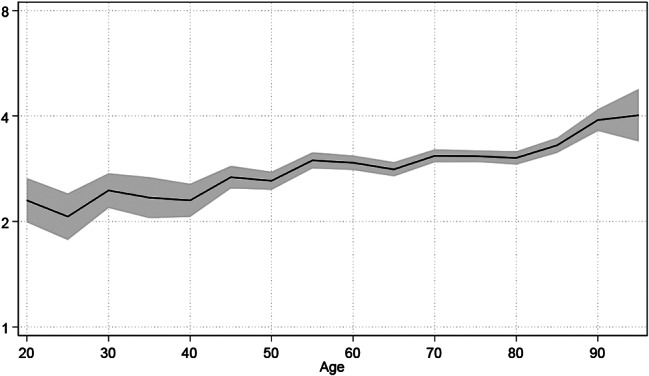
Analysis of the applicability of the reweighted Frailty Score in a not-only-elderly population in Freiburg *N* = 198,819

Applicability of the reweighted Frailty Score in a non-elderly population was assessed using all patients aged 18 years and older hospitalized between 2011 and 2014, *N* = 198,819. As the incidence of frailty is much lower in the younger population, a frailty score is unlikely to be used to support treatment decisions. However, such a score can be used for risk adjustment. Considering discrimination alone, the AUC of 0.92 [0.91–0.92] of the reweighted frailty score is significantly superior to that of the Charlson score (AUC = 0.76 [0.75–0.77]) and the original frailty score (AUC = 0.87 [0.87–0.88]) in this population as well. The relative influence of the reweighted frailty score on hospital mortality is shown in Fig. 4. Here we use odds ratios to show the influence of an increase in the reweighted frailty score on hospital mortality in individual age groups. Overall, it must be said that the influence is surprisingly uniform across the different age groups. If women and men are considered separately, a similar picture emerges: We observe a fairly age-stable influence of the reweighted frailty score on hospital mortality, which does not differ significantly for women and men.

## Discussion

Gilbert et al. [12] developed the Hospital Frailty Risk Score based on data from patients admitted to England’s National Health Service (NHS) hospitals. Its primary advantage lies in its reliance solely on ICD-10 codes, allowing its use wherever this coding system is in operation. Notably, this score can seamlessly integrate into hospital information systems, eliminating variability among operators and the burdens associated with manual scoring methods. To our knowledge, the present work is the first external validation of Gilbert’s Hospital Frailty Risk Score. Our approach to reweighting the Frailty Score resulted in substantially better discrimination than the initial weighting of the score. In addition, calibration curves show a good agreement between score-based predictions and actual observed mortality. Additional external validation using inpatient cases aged ≥ 75 in 2022 throughout Germany further underlines the geographic and temporal validity of the reweighted Frailty Score.

There are evident advantages in regularly identifying older individuals at risk of adverse outcomes during acute hospital stays. This identification facilitates tailored interventions for frailty starting from admission and continuing throughout the hospitalization. Other advantages encompass better service planning, resource allocation, and assessment specifically directed towards older individuals with frailty. The benefits of a country-specific frailty score are particularly relevant in the light of the German billing system of diagnosis-related groups (DRGs). The DRG system reimburses hospitals based on the specific diagnoses and procedures performed. This structure can create a financial incentive to perform more interventions or procedures, even if they may not be in the best interest of frail patients. Consequently, the DRG system may undervalue conservative, non-invasive treatments that are often more suitable for frail patients.

Moreover, a key area of application for the risk score is risk adjustment in observational studies. Billing data is often used to compare competing treatment methods [29]. In contrast to randomized studies, a simple direct comparison is not very meaningful here and great efforts must be made to establish comparability for groups that are not actually comparable [29]. Three aspects are usually used for this purpose: (1) disease-specific relevant comorbidities, (2) disease-specific risk scores and (3) cross-disease risk scores. The Charlson score is often used for the latter [30–33]. Due to the complexity of its collection, frailty is rarely used for risk adjustment in this context, although it can be extremely relevant [12]. And this is precisely where we believe that the frailty score presented here can be used. The frailty score also has advantages over general risk scores such as the Charlson score. As the Charlson score contains many internal and cardiovascular comorbidities, its use for cardiovascular diseases is sometimes problematic: it is always possible that a comorbidity only arises during the course of the stay, as an outcome of the treatment, so to speak. If this possibility exists, the respective risk score should not be used for risk adjustment. The frailty score, on the other hand, is only slightly affected by this circumstance, as frailty-related comorbidities can usually occur little or not at all in the course of an individual hospitalization.

There were a few research limitations. First, the main part of the presented research is a single-center study, the findings of which might not be generalizable. Although we were able to verify the results using a Germany-wide sample, the entire score development process is still associated with the limitations of a single-center study. Second, there are no time-stamps attached to the ICD-10 data. This complicates the external validity and generalizability of the reweighted Frailty Score in the following ways: It is very likely that not all frailty-related diagnoses are coded at admission of the patients. Instead, some aspects may only be noticed during the course of hospitalization and then documented using ICD-10 coding. As a result, the likelihood of an aspect finding its way into the coding is strongly correlated with the length of stay [34]. For example, a patient who is hospitalized for a medical emergency and dies shortly after admission has a very low probability that all aspects will be fully coded. As a result, the score can only ever be used if a complete patient history is available or a systematic anamnesis was carried out. In everyday clinical practice, this requirement is not always met, particularly in the case of emergency patients and/or short-stay patients, which is why the reweighted Frailty Score tends to underestimate the degree of frailty among these patients.

### Electronic supplementary material

Below is the link to the electronic supplementary material.

Supplementary Material 1

Supplementary Material 2

## Data Availability

The datasets analyzed during the current study are not publicly available due to German data protection regulations but are available from the corresponding author on reasonable request.

## References

[1] Kim DH (2020). Measuring frailty in health care databases for clinical care and research. Ann Geriatr Med Res.

[2] Bergman H, Ferrucci L, Guralnik J, Hogan DB, Hummel S, Karunananthan S (2007). Frailty: an emerging research and clinical paradigm—issues and controversies. J Gerontol Biol Sci Med Sci.

[3] Clegg A, Young J, Iliffe S, Rikkert MO, Rockwood K (2013). Frailty in elderly people. Lancet.

[4] Puts MTE, Lips P, Deeg DJH (2005). Sex differences in the risk of Frailty for Mortality Independent of Disability and Chronic diseases. J Am Geriatr Soc.

[5] Wallis SJ, Wall J, Biram RWS, Romero-Ortuno R (2015). Association of the clinical frailty scale with hospital outcomes. QJM Int J Med.

[6] Hajek A, Bock J-O, Saum K-U, Matschinger H, Brenner H, Holleczek B (2018). Frailty and healthcare costs—longitudinal results of a prospective cohort study. Age Ageing.

[7] Ahmed N, Mandel R, Fain MJ (2007). Frailty: an emerging geriatric syndrome. Am J Med.

[8] Chong E, Ho E, Baldevarona-Llego J, Chan M, Wu L, Tay L (2018). Frailty in hospitalized older adults: comparing different frailty measures in predicting short-and long-term patient outcomes. J Am Med Dir Assoc.

[9] Theou O, Brothers TD, Mitnitski A, Rockwood K (2013). Operationalization of Frailty using eight commonly used scales and comparison of their ability to Predict all-cause mortality. J Am Geriatr Soc.

[10] Rockwood K, Song X, MacKnight C, Bergman H, Hogan DB, McDowell I (2005). A global clinical measure of fitness and frailty in elderly people. CMAJ.

[11] McCusker J, Bellavance F, Cardin S, Trepanier S, Verdon J, Ardman O (1999). Detection of older people at increased risk of adverse Health outcomes after an emergency visit: the ISAR Screening Tool. J Am Geriatr Soc.

[12] Gilbert T, Neuburger J, Kraindler J, Keeble E, Smith P, Ariti C (2018). Development and validation of a hospital frailty risk score focusing on older people in acute care settings using electronic hospital records: an observational study. Lancet.

[13] McAlister F, van Walraven C (2019). External validation of the Hospital Frailty Risk score and comparison with the hospital-patient one-year Mortality Risk score to predict outcomes in elderly hospitalised patients: a retrospective cohort study. BMJ Qual Saf.

[14] Eckart A, Hauser SI, Haubitz S, Struja T, Kutz A, Koch D (2019). Validation of the hospital frailty risk score in a tertiary care hospital in Switzerland: results of a prospective, observational study. BMJ Open.

[15] McAlister FA, Lin M, Bakal JA (2019). Prevalence and Postdischarge Outcomes Associated with Frailty in Medical inpatients: impact of different Frailty definitions. J Hosp Med.

[16] Shebeshi DS, Dolja-Gore X, Byles J (2021). Validation of hospital frailty risk score to predict hospital use in older people: evidence from the Australian longitudinal study on women’s Health. Arch Gerontol Geriatr.

[17] Keeble E, Roberts HC, Williams CD, Van Oppen J, Conroy SP (2019). Outcomes of hospital admissions among frail older people: a 2-year cohort study. Br J Gen Pract.

[18] Kwok CS, Zieroth S, Van Spall HG, Helliwell T, Clarson L, Mohamed M (2020). The Hospital Frailty Risk score and its association with in-hospital mortality, cost, length of stay and discharge location in patients with heart failure short running title: Frailty and outcomes in heart failure. Int J Cardiol.

[19] Kwok CS, Lundberg G, Al-Faleh H, Sirker A, Van Spall HG, Michos ED (2019). Relation of frailty to outcomes in patients with acute coronary syndromes. Am J Cardiol.

[20] McAlister FA, Savu A, Ezekowitz JA, Armstrong PW, Kaul P (2020). The hospital frailty risk score in patients with heart failure is strongly associated with outcomes but less so with pharmacotherapy. J Intern Med.

[21] Hannah TC, Neifert SN, Caridi JM, Martini ML, Lamb C, Rothrock RJ (2020). Utility of the hospital frailty risk score for predicting adverse outcomes in degenerative spine surgery cohorts. Neurosurgery.

[22] Bruno RR, Wernly B, Flaatten H, Schölzel F, Kelm M, Jung C (2019). The hospital frailty risk score is of limited value in intensive care unit patients. Crit Care.

[23] von der Warth R, Hehn P, Wolff J, Kaier K (2020). Hospital costs associated with post-traumatic stress disorder in somatic patients: a retrospective study. Health Econ Rev.

[24] Zou H (2006). The adaptive Lasso and its Oracle Properties. J Am Stat Assoc.

[25] Tibshirani R (1996). Regression shrinkage and selection via the lasso. J R Stat Soc Ser B Stat Methodol.

[26] DeLong ER, DeLong DM, Clarke-Pearson DL. Comparing the areas under two or more correlated receiver operating characteristic curves: a nonparametric approach. Biometrics. 1988;:837–45. 3203132

[27] Steyerberg EW, Vergouwe Y (2014). Towards better clinical prediction models: seven steps for development and an ABCD for validation. Eur Heart J.

[28] Austin PC, Steyerberg EW (2014). Graphical assessment of internal and external calibration of logistic regression models by using loess smoothers. Stat Med.

[29] Stachon P, Kaier K, Zirlik A, Bothe W, Heidt T, Zehender M et al. Risk-adjusted comparison of In‐Hospital outcomes of Transcatheter and Surgical aortic valve replacement. J Am Heart Assoc. 2019;8. 10.1161/JAHA.118.011504 PMC6509703 30897991

[30] Maier A, Kaier K, Heidt T, Westermann D, von zur Mühlen C, Grundmann S (2023). Catheter based left atrial appendage closure in-hospital outcomes in Germany from 2016 to 2020. Clin Res Cardiol.

[31] Haverkamp C, Kaier K, Shah M, von Zur Mühlen C, Beck J, Urbach H et al. Cerebral aneurysms: Germany-wide real-world outcome data of endovascular or neurosurgical treatment from 2007 to 2019. J NeuroInterventional Surg. 2023. 10.1136/jnis-2023-020181 PMC10958314 37290919

[32] Roth K, Kaier K, Stachon P, von zur Mühlen C, Jungmann P, Grimm J (2023). Evolving trends in the surgical therapy of patients with endometrial cancer in Germany: analysis of a nationwide registry with special emphasis on perioperative outcomes. Arch Gynecol Obstet.

[33] Engler-Hüsch S, Heister T, Mutters NT, Wolff J, Kaier K (2018). In-hospital costs of community-acquired colonization with multidrug-resistant organisms at a German teaching hospital. BMC Health Serv Res.

[34] Wolff J, Heister T, Normann C, Kaier K (2018). Hospital costs associated with psychiatric comorbidities: a retrospective study. BMC Health Serv Res.

