# Supplementary material for: Reweighting and validation of the hospital frailty risk score using electronic health records in Germany: a retrospective observational study
Source: BMC Geriatr. 2024 Jun 13;24:517. doi: 10.1186/s12877-024-05107-w (PMC11177354; doi:10.1186/s12877-024-05107-w)
Supplement: Supplementary file 1 — Supplementary Material 1 [file 12877_2024_5107_MOESM1_ESM.docx]

**Table S1:** List of 53 comorbidities that were selected within the score development process, frequency in the development cohort in Freiburg (admission year 2011 to 2013, N=30,525) and the validation cohorts in Freiburg (admission year 2014, N=11,202) and Germany (admission year 2022, N=491,251) and number of points awarded for each to create the reweighted Frailty Score

| **ICD Description and code** | **N=30,525** |  | **N=11,202** |  | **N=491,251** | **Points**  **awarded** |
| --- | --- | --- | --- | --- | --- | --- |
| Calculus of kidney and ureter (N20) | 0.34% |  | 0.39% |  | 0.34% | -2.1 |
| Respiratory failure, not elsewhere classified (J96) | 9.93% |  | 10.23% |  | 13.41% | 1.8 |
| Somnolence, stupor and coma (R40) | 5.13% |  | 5.44% |  | 2.50% | 1.7 |
| Other septicaemia (A41) | 2.02% |  | 2.49% |  | 1.49% | 1.5 |
| Acute renal failure (N17) | 5.41% |  | 5.99% |  | 7.32% | 1.3 |
| Other disorders of kidney and ureter, not elsewhere classified (N28) | 0.68% |  | 0.53% |  | 1.05% | -1.2 |
| Deficiency of other B group vitamins (E53) | 0.40% |  | 0.31% |  | 3.24% | -1.2 |
| Blindness and low vision (H54) | 6.81% |  | 5.37% |  | 1.09% | -1.2 |
| Personal history of other diseases and conditions (Z87) | 0.36% |  | 0.40% |  | 1.34% | -1.1 |
| Symptoms and signs involving emotional state (R45) | 0.40% |  | 0.73% |  | 1.05% | 1.0 |
| Mental and behavioural disorders due to use of alcohol (F10) | 0.86% |  | 0.98% |  | 0.49% | -0.9 |
| Spinal stenosis (M48) | 2.54% |  | 2.58% |  | 2.18% | -0.9 |
| Symptoms and signs concerning food and fluid intake (R63) | 2.89% |  | 2.91% |  | 2.96% | 0.9 |
| Intracranial injury (S06) | 2.96% |  | 3.13% |  | 2.45% | 0.9 |
| Pneumonitis due to solids and liquids (J69) | 1.72% |  | 1.81% |  | 1.30% | 0.9 |
| Cerebral Infarction (I63) | 4.66% |  | 4.63% |  | 3.10% | 0.9 |
| Artificial opening status (Z93) | 1.66% |  | 1.80% |  | 1.12% | -0.8 |
| Other disorders of pancreatic internal secretion (E16) | 0.15% |  | 0.29% |  | 0.22% | 0.7 |
| Fever of unknown origin (R50) | 1.94% |  | 1.54% |  | 1.29% | -0.7 |
| Other disorders of urinary system (N39) | 6.85% |  | 7.70% |  | 14.74% | -0.7 |
| Osteoporosis without pathological fracture (M81) | 2.26% |  | 2.43% |  | 3.99% | -0.7 |
| Other joint disorders, not elsewhere classified (M25) | 0.41% |  | 0.61% |  | 1.07% | -0.7 |
| Other cerebrovascular diseases (I67) | 3.91% |  | 6.81% |  | 2.74% | -0.6 |
| Decubitus ulcer (L89) | 5.46% |  | 5.15% |  | 4.03% | 0.6 |
| Transient cerebral ischaemic attacks and related syndromes (G45) | 1.79% |  | 1.78% |  | 1.09% | -0.6 |
| Dependence on enabling machines and devices (Z99) | 3.18% |  | 2.56% |  | 2.85% | -0.6 |
| Other symptoms and signs involving general sensations and perceptions (R44) | 0.14% |  | 0.15% |  | 0.26% | -0.6 |
| Other hearing loss (H91) | 3.27% |  | 3.37% |  | 2.95% | -0.5 |
| Other diseases of digestive system (K92) | 1.83% |  | 1.85% |  | 1.97% | 0.5 |
| Fracture of rib(s), sternum and thoracic spine (S22) | 1.13% |  | 1.29% |  | 1.53% | 0.5 |
| Other and unspecified injuries of head (S09) | 0.08% |  | 0.09% |  | 0.02% | 0.5 |
| Unspecified dementia (F03) | 2.80% |  | 2.80% |  | 7.94% | -0.4 |
| Unspecified urinary incontinence (R32) | 4.74% |  | 2.95% |  | 7.44% | -0.4 |
| Pneumonia, organism unspecified (J18) | 4.27% |  | 4.00% |  | 4.70% | 0.4 |
| Epilepsy (G40) | 2.83% |  | 2.89% |  | 2.48% | -0.4 |
| Complications of genitourinary prosthetic devices, implants and grafts (T83) | 0.43% |  | 0.68% |  | 0.72% | -0.4 |
| Abnormalities of gait and mobility (R26) | 6.51% |  | 6.00% |  | 10.93% | 0.4 |
| Convulsions, not elsewhere classified (R56) | 0.59% |  | 0.75% |  | 0.17% | 0.4 |
| Dysphagia (R13) | 2.69% |  | 2.83% |  | 3.20% | -0.3 |
| Vascular dementia (F01) | 1.24% |  | 0.99% |  | 1.95% | 0.3 |
| Thyrotoxicosis [hyperthyroidism] (E05) | 2.60% |  | 2.10% |  | 1.55% | -0.3 |
| Retention of urine (R33) | 1.67% |  | 1.67% |  | 1.60% | -0.3 |
| Problems related to care-provider dependency (Z74) | 10.96% |  | 9.27% |  | 20.68% | -0.3 |
| Other disorders of fluid, electrolyte and acid-base balance (E87) | 12.83% |  | 14.45% |  | 22.25% | 0.2 |
| Unspecified haematuria (R31) | 1.87% |  | 2.01% |  | 2.01% | -0.2 |
| Sequelae of cerebrovascular disease (secondary codes) (I69) | 3.51% |  | 3.20% |  | 2.93% | -0.2 |
| Speech disturbances, not elsewhere classified (R47) | 6.21% |  | 6.21% |  | 4.19% | 0.2 |
| Open wound of head (S01) | 1.44% |  | 1.60% |  | 2.10% | -0.2 |
| Other functional intestinal disorders (K59) | 3.80% |  | 2.65% |  | 7.97% | 0.1 |
| Other symptoms and signs involving the nervous and musculoskeletal systems (R29) | 9.69% |  | 6.85% |  | 5.74% | -0.1 |
| Abnormalities of heart beat (R00) | 2.02% |  | 1.52% |  | 1.86% | 0.1 |
| Other noninfective gastroenteritis and colitis (K52) | 0.67% |  | 0.46% |  | 1.00% | -0.1 |
| Hemiplegia (G81) | 5.25% |  | 4.72% |  | 3.63% | 0.1 |
