# Supplementary material for: Reweighting and validation of the hospital frailty risk score using electronic health records in Germany: a retrospective observational study
Source: BMC Geriatr. 2024 Jun 13;24:517. doi: 10.1186/s12877-024-05107-w (PMC11177354; doi:10.1186/s12877-024-05107-w)
Supplement: Supplementary file 2 — Supplementary Material 2 [file 12877_2024_5107_MOESM2_ESM.docx]

**Figure S1:** Calibration plots in the validation cohorts in Freiburg (A, B and C, N=11,202) and Germany (D, E and F, N=491,251)

| A) Charlson Score, N=11,202   | B) original Frailty Score, N=11,202   | C) reweighted Frailty Score, N=11,202   |
| --- | --- | --- |
| D) Charlson Score, N=491,251   | E) original Frailty Score, N=491,251   | F) reweighted Frailty Score, N=491,251   |

Figure legend: In the validation cohorts, the observed risk of in-hospital mortality is plotted against the predicted risk from the Charlson Score, the original Frailty Score and the reweighted Frailty Score. The solid line represents perfect calibration (with a slope of 1), and the dashed line represents the respective loess smoothed calibration curves. Each grouping of predicted risk is represented with an open circle along this calibration curve with 95% confidence intervals (CIs). When the Lowess line is below the dashed line of perfect calibration, this suggests overestimation of outcomes. Relative frequencies of the predicted values of the three scores are shown at the top of the figures.

**Figure S2:** Analysis of the applicability of the reweighted Frailty Score in a not-only-elderly population in Freiburg N=198,819

| A) female patients (N=96,771)   | B) male patients (N=102,048)   |
| --- | --- |
